# Numerical Study on the Evaporation of a Non-Spherical Sessile Droplet

**DOI:** 10.3390/mi14010076

**Published:** 2022-12-28

**Authors:** Wenbin Cui, Yang Cao, Shoupei Wang, Tianci Zhang, Hongbin Ma, Chao Chang, Dalong Liang, Jingming Dong

**Affiliations:** 1 College of Marine Engineering, Dalian Maritime University, Dalian 116016, China; 2 Department of Mechanical and Aerospace Engineering, University of Missouri, Columbia, MO 65211, USA

**Keywords:** numerical simulation, non-spherical sessile droplet, evaporation

## Abstract

To better understand the evaporation of a non-spherical droplet, a two-dimensional simulation was conducted to investigate the evaporation on the asymmetric cross-section of non-spherical sessile droplets, which are characterized by two curvatures with two different contact angles on both sides. The temperature distribution, internal flow, and evaporation flux distribution at a quasi-steady state were revealed to be different from the spherical droplets. When heated from the substrate, the lowest surface temperature moves to the side of higher curvature or larger contact angle, forming a single vortex in the droplet. This single-vortex formation continues to be enhanced by enlarging the contact angle discrepancy. Unlike spherical droplets, the smaller curvature side of a non-spherical sessile droplet will release more evaporation flux. In addition, it is found that the non-spherical sessile droplets could surpass the spherical sessile droplets in evaporation flux.

## 1. Introduction

Evaporation of a sessile droplet has extensive applications, which can be found in painting [1], inkjet printing [2], droplet cooling [3,4], medical testing [5], and DNA extraction [6]. Evaporation behavior, evaporation rate, and internal flow are the primary focus of most sessile droplet evaporation studies. The evaporation behavior of a droplet can have three basic modes, i.e., the constant contact angle mode, the constant contact area mode, and the mixed mode [7], and these modes are mainly determined by the pinning force resulting from surface roughness [8,9].

The evaporation rate of a sessile droplet has a great relationship with surface wettability; therefore, a theoretical analysis was conducted to predict the evaporation rate considering the contact angle. Picknett and Bexon [10] proposed a function based on the analogy between the electrostatic potential and the diffusive flux for calculating the evaporation rate of a sessile droplet, as:

(1) $$-\frac{dm}{dt}=2\pi rD\Delta Cf\left(\theta \right)$$

where *r* is the radius of the sessile droplet and *f*(*θ*) is:

$$f\left(\theta \right)=\{\begin{array}{c} 0.6366\theta +0.09591\theta^{2}-0.06144\theta^{3}\left(\theta <\pi /18\right)\\ 0.00008957+0.6333\theta +0.116\theta^{2}-0.08878\theta^{3}+0.01033\theta^{4}\left(\theta \geq \pi /18\right) \end{array}$$

Thereafter, Hu and Larson [11] simplified *f*(*θ*) in Picknett and Bexon’s function [10] as: *f* (*θ*) = 0.27*θ*^2^ + 1.3, and the calculated results agreed well with the experimental results for any initial contact angle, *θ,* between 0 and π/2. Rowan et al. [12] supposed the height of the droplet and the contact angle were approximately linear in time in the regime of the constant contact area and the rate of mass loss to be proportional to the height, which can be expressed as:

(2) $$-\frac{dm}{dt}=2\pi D\Delta Ch$$

where *h* is the height of the droplet.

Although the analytical formulae have been proven accurate in predicting the evaporation rate of a sessile droplet, the analytical solutions are only feasible for the occasions with simplifying assumptions, including spherical droplet profile, quasi-steady vapor diffusion process, and isothermal droplet [13]. Numerical simulation does not require these assumptions and therefore can describe more complicated temperature distributions and resultant Marangoni flow in the droplets [14]. As well as the application of conservation equations, VOF (volume of fluid) [15], LS (level set) [16], or arbitrary Lagrangian–Eulerian formulation [17] methods could also be combined to better correlate the processes of heat transfer and the mass transfer by tracing the liquid–air interface during the evolution of the droplet volume. Moreover, the lattice Boltzmann method is also a remarkable tool for the simulation [18] since it could trace the interface as well.

For a real droplet, to study the internal flow, which is usually induced by the capillary force, i.e., the Marangoni effect, particle tracing is an effective method commonly used in the research. By studying the evaporation of a colloid droplet, Deegan et al. [19] explained the formation of the capillary flow in evaporating droplets. By detecting a non-uniformly heated colloid droplet, a symmetric Marangoni recirculation was found resulted from the symmetric distribution of the temperature on the liquid–air interface [20]. As exhibited by the behavior of the particles, the Marangoni flows were reported to become unstable in surfactant-laden water droplets, and local vortex cells were observed along the contact line, which contributed a pumping force, pushing the fluid outward from the droplet [21].

**Figure 1:**
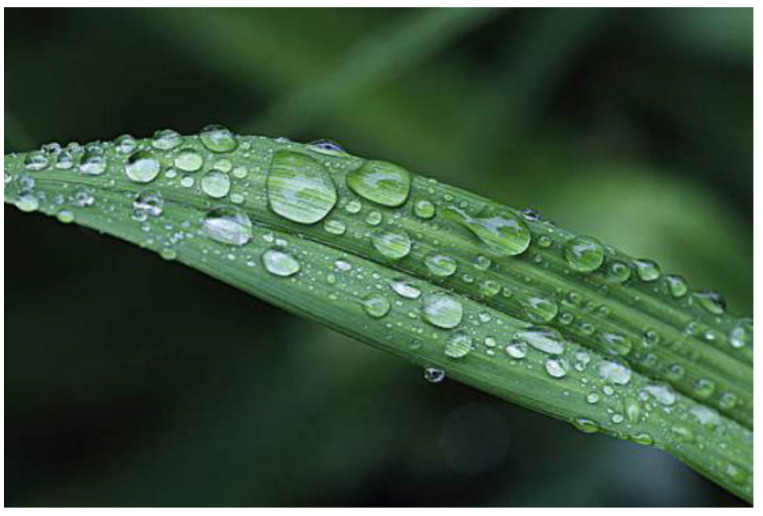
Non-spherical droplets on a grass leaf.

In most studies on the sessile droplet evaporation, the sessile droplet is spherical or assumed to be spherical. However, it is clear that the structure on the solid surface is commonly heterogeneous in nature, and most sessile droplets appear to be non-spherical due to the nonuniform structure formed on the surface, as shown in Figure 1. Therefore, researchers have often referred to the shape effect on the evaporation process in their recent works. Sáenz et al. [22] revealed the surface temperature distribution and internal flow during the evaporation process of a non-axisymmetric droplet by experiment and numerical simulation. Their results showed that the surface temperature was distributed non-axisymmetrically, and the liquid in the droplet was shown to flow from one side to the other side, resulting in vortexes forming on the side of the higher curvature. Wang and Shi [23] investigated the shape effect on the hydrothermal wave distribution in non-spherical droplets. They concluded that cells responding to the Bénard–Marangoni instability occurred first at the region where the curvature of the three-phase contact line was the largest and always drifted from the lower curvature regions toward the higher ones. Cui et al. [24] reported the evaporation process of a non-axisymmetric sessile droplet on a hybrid structured surface consisting of half hydrophilicity and half hydrophobicity. The non-axisymmetric shape induced a higher evaporation rate on the hydrophobic half, where the curvature of the droplet liquid–air (LA) interface was lower.

The non-spherical shape could lead to the droplet evaporation being different from the spherical droplet. So far, few publications on non-spherical droplet evaporation are available, and further research is needed to reveal its working principle. In this study, a 2D simulation was conducted to investigate evaporation on an asymmetric cross-section of non-spherical sessile droplets, which is a vital and often overlooked factor affecting the asymmetric evaporation process. The effects of heating temperature and the contact angle discrepancy (CAD) are analyzed for a better understanding of the evaporation principle.

## 2. Numerical Simulation

**Figure 2:**
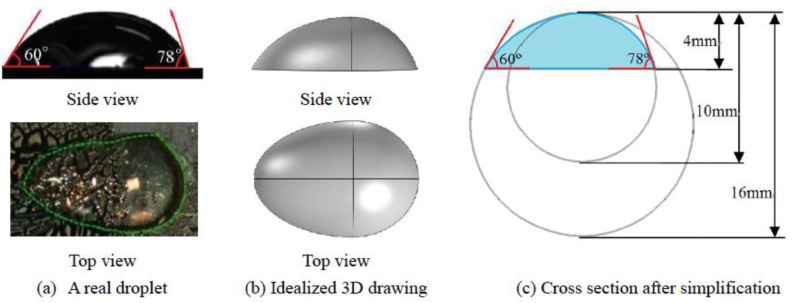
The cross-section of a non-spherical droplet derived from a real droplet. (**a**) is the top view and the side view of a real droplet resting on a hybrid structured MWCNT surface; (**b**) is an idealized 3D drawing of the real droplet (**a**) by composing part of a sphere and part of an ellipsoid; Based on (**b**), the non-spherical droplet cross section was further idealized by composing parts of two circles (**c**), which would be studied in this work.

Figure 2 shows the cross-section of a ~10 μL droplet resting on a hybrid structured multiwalled carbon nanotube (MWCNT) surface, which was derived from a real non-spherical droplet investigated by Cui et al. [24] (Figure 2a). The droplet seen in 3D (Figure 2b) was composed of part of a sphere and part of an ellipsoid. However, the curvature of the ellipsoid was not uniform on the liquid–air interface, and the resultant analysis would be complicated by considering the curvature effect. Considering the shape of a real non-spherical droplet, the cross-section of the droplet, as seen from a side view in Figure 2b, can be represented by parts of two circles, with radii of 8 and 5 mm, respectively, and the corresponding contact angle (CA) on either side is 60° and 78°, as shown in Figure 2c.

In addition, the following assumptions are made:

(1) The surface of the silicon substrate is assumed to be smooth.

(2) The liquid–air interface revolution is not included in the simulation since the mesh is set as fixed, otherwise the shape of the non-spherical droplet would turn into the shape of a spherical droplet as the simulation proceeds.

(3) Both fluids (air and water) are assumed to be incompressible.

### 2.1. Governing Equations

Momentum and continuity equations in liquid and air domains can be expressed as:

(3) $$\rho \left(\frac{\partial u}{\partial t}+u\frac{\partial u}{\partial x}+v\frac{\partial u}{\partial y}\right)=-\frac{\partial p}{\partial x}+\mu \left(\frac{\partial u^{2}}{\partial^{2}x}+\frac{\partial u^{2}}{\partial^{2}y}\right)$$

(4) $$\rho \left(\frac{\partial v}{\partial t}+u\frac{\partial v}{\partial x}+v\frac{\partial v}{\partial y}\right)=-\frac{\partial p}{\partial y}+\mu \left(\frac{\partial v^{2}}{\partial^{2}x}+\frac{\partial v^{2}}{\partial^{2}y}\right)+\rho g$$

(5) $$\frac{\partial u}{\partial x}+\frac{\partial v}{\partial y}=0$$

The vapor transfer in the air domain is controlled by:

(6) $$\frac{\partial c}{\partial t}+u\frac{\partial c}{\partial x}+v\frac{\partial c}{\partial y}=D\left(\frac{\partial^{2}c}{\partial x^{2}}+\frac{\partial^{2}c}{\partial y^{2}}\right)$$

The heat transfer in the liquid and air domains can be expressed as:

(7) $$\rho C_{p}\left(\frac{\partial T}{\partial t}+u\frac{\partial T}{\partial x}+v\frac{\partial T}{\partial y}\right)=k\left(\frac{\partial^{2}T}{\partial x^{2}}+\frac{\partial^{2}T}{\partial y^{2}}\right)$$

The heat transfer in the solid domain is given as:

(8) $$\rho C_{p}\frac{\partial T}{\partial t}=k\left(\frac{\partial^{2}T}{\partial x^{2}}+\frac{\partial^{2}T}{\partial y^{2}}\right)$$

### 2.2. Boundary Conditions

The initial temperature of liquid, air, and solid is set at the ambient temperature of 293.15 K, the initial vapor concentration in the air is set at an ambient concentration of 0.2 mol/m^3^, and the initial velocity in liquid and air is set at 0 m/s. At the outer boundary of the air far away from the droplet, the temperature is kept constant at the ambient temperature, and the vapor concentration is kept constant at the ambient concentration.

At the liquid–air interface, the stresses are balanced and expressed as:

(9) $$n\cdot (\tau_{v}-\tau_{l})=f_{st}$$

where *τ_v_* and *τ_l_* are the total stress tensors in the vapor phase and liquid phase at the interface, respectively, ***n*** is the unit vector normal to the interface, and *f_st_* is the force per unit area due to the surface tension. The components of *f_st_* can be either normal or tangential to the boundary. In the normal direction, the force balance is:

(10) $$n\cdot (\tau_{v}-\tau_{l})\cdot n=\frac{\sigma }{r_{c}}\cdot n$$

where *r_c_* is the curvature radius. In the tangential direction, the force balance is:

(11) $$n\cdot (\tau_{v}-\tau_{l})\cdot t=\left(\frac{\partial \sigma }{\partial x}+\frac{\partial \sigma }{\partial y}\right)\cdot t$$

where ***t*** is the tangential vector of the interface. The right term in the above equation is the thermocapillary stress due to surface temperature difference, which causes the Marangoni flow.

The water–air interface surface tension in Equation (11) is influenced by temperature and shown as [25]:

(12) $$\sigma =-2.3519705\cdot 10^{-7}T_{surf}{}^{2}-1.63350014\cdot 10^{-5}T_{surf}+9.77001279\cdot 10^{-2}$$

The vapor concentration at the interface is assumed to be saturated and can be calculated as:

(13) $$c_{surf}=c_{sat}\left(T_{surf}\right)=\frac{p_{v,sat}}{RT_{surf}}$$

in which, *p_v,sat_* can be expressed as [26]:

(14) $$p_{v,sat}[\mathrm{MPa}]=\exp\left(9.487-\frac{3.893\times 10^{3}}{T_{surf}[{}^{\circ}C]+230.47}\right)$$

The evaporation flux, *J,* caused by evaporation through the liquid–air interface is given as:

(15) $$J=-D\left(\frac{\partial c}{\partial x}+\frac{\partial c}{\partial y}\right)$$

where the diffusion coefficient, *D,* is set as 2.6 × 10^−5^m^2^/s.

The local mass loss of liquid is calculated by:

(16) $$\overset{•}{m}=JM_{r}$$

The evaporation cooling effect on the droplet can be given as:

(17) $$k_{l}\left(\frac{\partial T}{\partial x}+\frac{\partial T}{\partial y}\right)=\overset{•}{m}h_{lv}$$

Finally, the sides of the substrate are assumed to be adiabatic.

**Figure 3:**
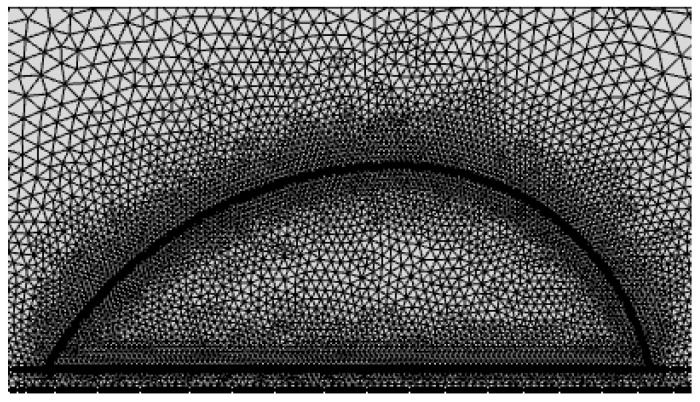
Typical mesh in the water domain.

**Figure 4:**
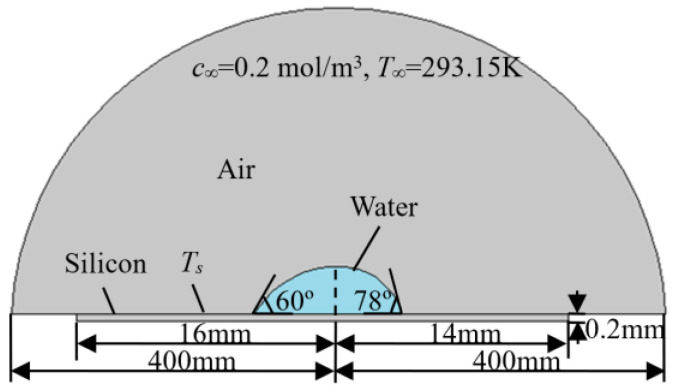
The geometry of the domains.

The numerical simulation was conducted by the finite element method, using the software program COMSOL Multiphysics 5.2. Modules of heat transfer and transport of diluted species were selected with laminar two-phase flow for discretization. Heat transfer, mass transfer, and velocity distribution during the evaporation process can be simulated for the droplet shown in Figure 2c with the given conditions indicated above. Figure 3 shows the mesh grid to be employed for the simulation, with a uniform mesh grid of 0.1 mm at the air–liquid, air–solid, and liquid–solid interfaces. After the grid-independence tests, a mesh with 24,117 grids was adopted and the typical mesh in the water domain is shown in Figure 3. The radius of the air domain was selected as 400 mm after the air domain independence tests, as shown Figure 4.

A time-dependent solver was chosen to simulate the quasi-steady evaporation process, and the step for the calculation was selected as 0.1 s after testing at 0.01, 0.02, 0.05, and 0.2 s. The tolerance was set at 0.01 for all variables. The quasi-steady state was supposed to be achieved at 45 s when the variation ratio of the surface average temperature became smaller than 10^−5^ [17], and can be calculated as:

(18) $$\delta =\left(T_{av.t+1s}-T_{av.t}\right)/T_{av.t}$$

## 3. Results and Discussion

**Figure 5:**
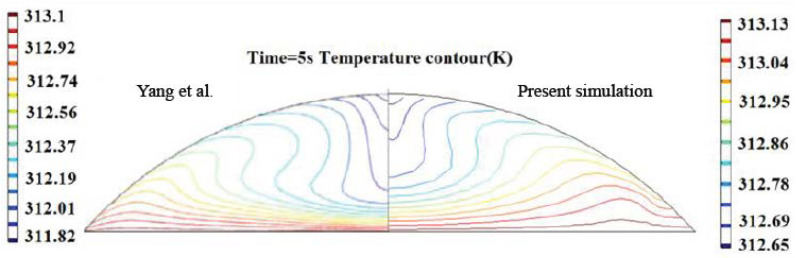
Present simulation results compared with Yang et al.’s work [17].

To verify the 2D simulation conducted herein, the evaporation of a spherical droplet heated at 313.15 K was simulated. With this temperature, a latent heat of vaporization of 2254 kJ/kg and a thermal conductivity of 0.64 W/(mK) were utilized for the calculation. Compared with that developed by Yang et al. [17], as shown in Figure 5, despite the shape difference between the 2D and 2D axisymmetric physical models, the temperature in the 2D simulation shows a similar distribution to their 2D axisymmetric simulation results.

### 3.1. The Heating Effect

**Figure 6:**
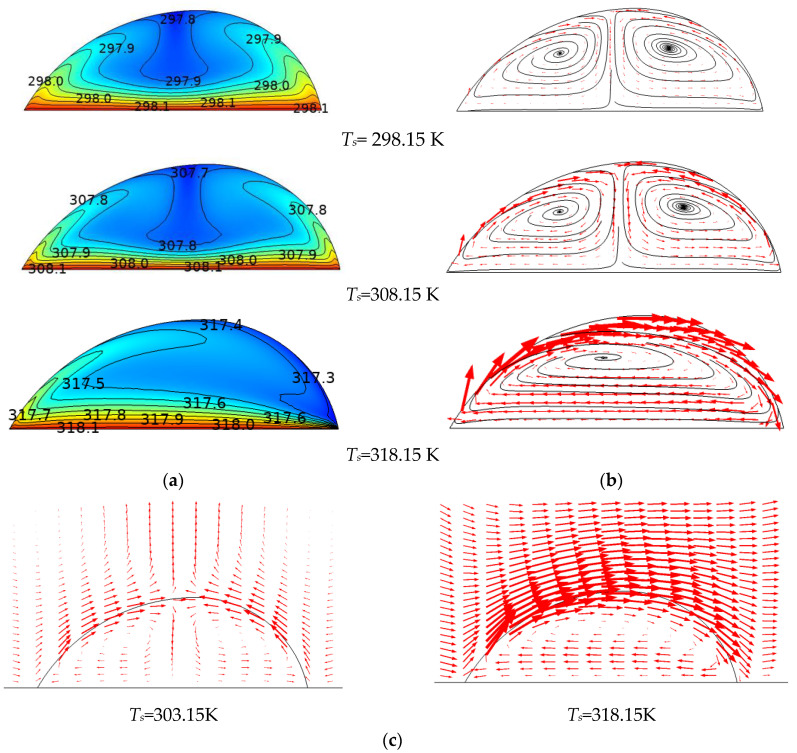
The evolution of the temperature and velocity at different substrate temperatures. The temperature and the velocity distribute apart in the droplet when *T_s_* = 298.15 K and 308.15 K, and a continuous distribution of temperature and a single vortex flow form when *T_s_* = 318.15 K, as shown in (**a**,**b**). The velocity distribution in the air acts correspondingly with the flow in the droplet, as shown in (**c**).

By setting the temperature of the substrate bottom from 298.15 to 323.15 K, the heating effect on the evaporation process of a non-spherical sessile droplet was simulated. As shown in Figure 6a, when the heating temperature was lower than 318.15 K, the lowest surface temperature appeared at the middle of the surface length. With the heating temperature raised to 318.15 K, the lowest temperature on the interface could no longer stay in the same position but moved to the side with a contact angle of 78°. This phenomenon could also be seen from the flow evolution in Figure 6b, in which the length of the arrows was proportional to the velocity. The lowest surface temperature could be viewed as a dividing point for the internal flow. With that flow’s invasive movement, the liquid was pushed toward the side of 78° until finally only one vortex existed in the droplet. The air flow acted correspondingly and passed over the droplet when the substrate was heated over 318.15 K, as shown in Figure 6c.

**Figure 7:**
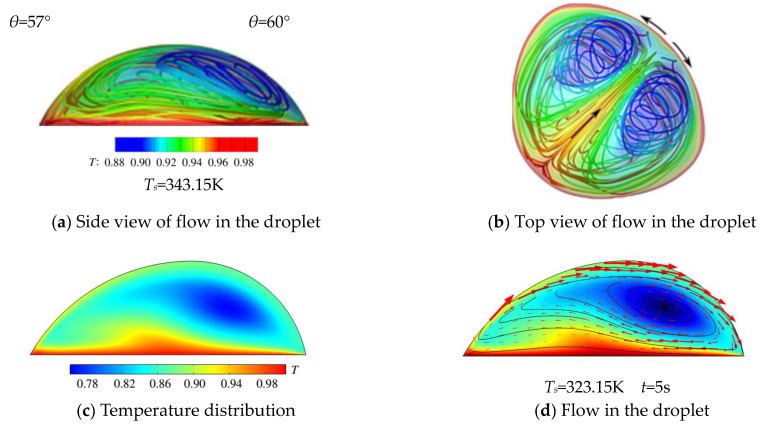
A comparison of the results from Sáenz et al.’s work [22] (**a,b**) and the present simulation (**c**,**d**). (The results of the present 2D simulation work, showing a lower temperature (**c**) and a single vortex (**d**) resting on the right side of the droplet, which were similar to Sáenz et al.’s work (**a**,**b**).

The single-vortex flow had been reported by Sáenz et al. [22]. In their work, an irregular sessile droplet of 57°–60° was heated at 343.15 Kand the surface temperature onthe side with the smaller contact angle of 57° is higher than the opposite side with the bigger contact angle of 60°. Based on their 3D transient simulation, the flow inside the droplet could be judged as originating from the side of 57°and running to the side of 60°, as shown in Figure 7a,b. Similarly, the present 2D simulation results also showed that the higher temperature resided on the side with the smaller contact angle when *T_s_* ≥ 318.15 K, as seen in Figure 7c; moreover, the flow started from this side as well, as illustrated in Figure 7d.

**Figure 8:**
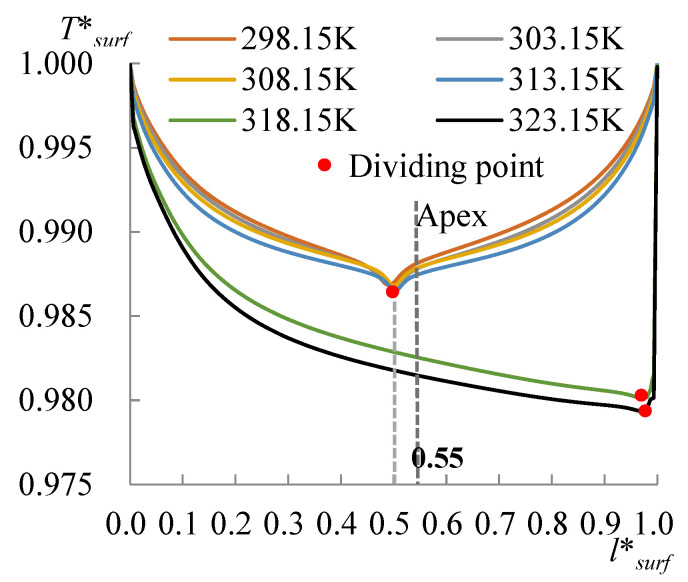
Temperature at the LA interface. For *T_s_* ≤ 313.15 K, the lowest surface temperature (dividing point) lies near the middle position rather than on the apex of the non-spherical droplet, and when *T_s_* = 318.15 K and 323.15 K, the lowest temperature moves to the rightmost side of the droplet.

**Figure 9:**
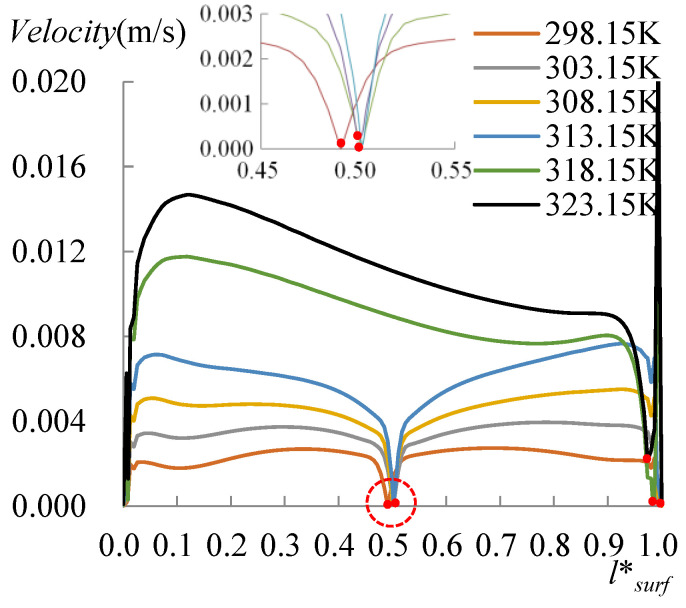
Velocity at the LA interface. There also exist dividing points of the velocity near the middle position of the surface length as surface temperatures when *T_s_* ≤ 313.15 K. The points moved rightward when *T_s_* = 298.15 to 303.13 K, and to the rightmost side of the droplet when *T_s_* ≥ 318.15 K.

**Figure 10:**
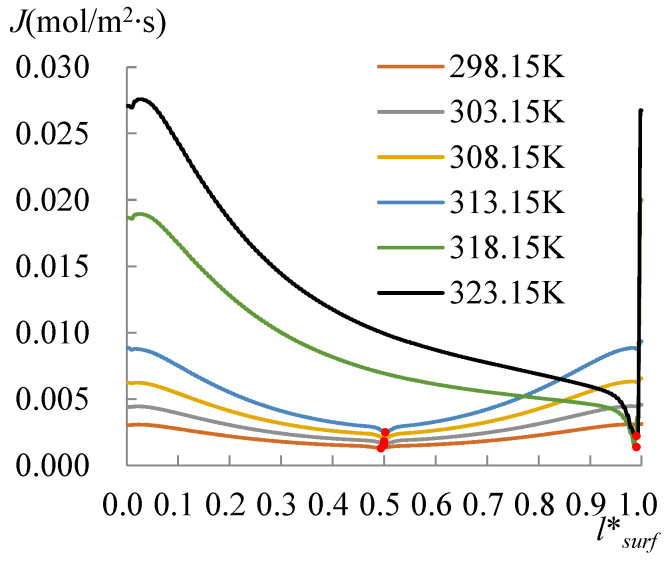
Evaporation flux at the LA interface. Evaporation flux is proportional to the local surface temperature; hence, a similar trend of its distribution is shown as the temperature distribution.

**Figure 11:**
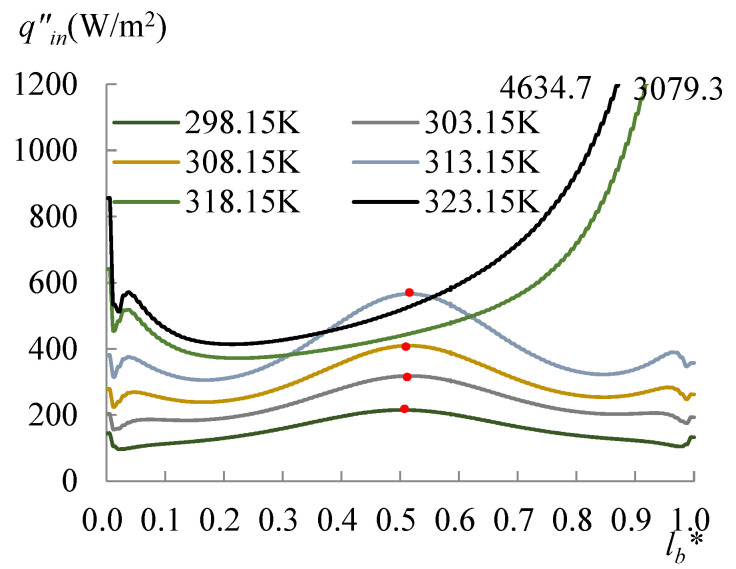
Heat flux conducted from the substrate. The heat conducted from the substrate is inversely proportional to the surface temperature of the droplet, however the dividing points still follow those of the surface temperature.

The evolution of the surface temperature with the heating temperature was evaluated with the dimensionless surface temperature, T*_surf_ = (*T_surf_* − 273.15 K)/(*T_s_* − 273.15 K), where *T_surf_* is the surface temperature on the liquid droplet, and *T_s_* is the temperature at the bottom of the substrate. Figure 8 shows the dimensionless surface temperature variation along l*_surf_, which is defined by *l*/*l_surf_*, where *l* is the curve length starting from the side with a CA of 60°, and *l_surf_* is the overall length of the LA interface. Clearly, the lowest surface temperature moved to the side with a CA of 78° when *T_s_* arrived at 318.15 K. The velocity and evaporation flux along the LA interface, and the heat flux conducted from the substrate, acted correspondingly with the increasing substrate temperature, as illustrated in Figure 9, Figure 10 and Figure 11. l*_b_ in Figure 11 is defined by *l*/*l_b_*, where *l* is the base length of the droplet that started from the side with a CA of 60° and *l_b_* is the overall length of the base length. As seen from Figure 9, the velocity also has dividing points, and these points stand near the middle position of the surface length, as for the surface temperature when the heating temperature is lower; however, there is a difference from the dividing points of temperatures for *T_s_* between 298.15 and 308.15 K, and there is a rightward movement with the *T_s_* enhancement in this range. After the substrate temperature was enhanced over 313.15 K, the dividing points moved to the rightmost ones of the surface temperature. Evaporation flux is proportional to the local surface temperature; hence, a similar trend of its distribution is shown in Figure 10. It can be noted that after the substrate temperature was enhanced over 313.15 K resulting in the formation of the single-vortex flow, the evaporative flux was much higher on the left side than on the right side. On the contrary, after the formation of the single-vortex flow at *T_s_* ≥ 318.15 K, the heat conducted from the substrate, which is inversely proportional to the surface temperature of the droplet, achieved the maximum as 4634.7 W/m^2^ on the right side of the droplet, as shown in Figure 11.

When the droplet evaporation arrives at a quasi-steady state, the power balance is assumed to be met and can be analyzed as:

(19) $$q_{in}-q_{out}=q_{internal}+q_{kinetic}$$

where *q_in_* is the input power conducted from the substrate, and *q_out_* is the output power, described as:

(20) $$q_{out}=q_{evap}+q_{cond}$$

in which *q_evap_* is the power lost due to evaporation, and *q_cond_* is the power lost due to conduction from the LA interface to the air. The right side of Equation (19) represents the power left in the droplet, which consisted of the internal power, *q_internal_,* and the kinetic power, *q_kinetic_*. In the 2D simulation, power is described as the heat flow, calculated by:

(21) $$q=\bar{q\prime \prime }l$$

where $\bar{q\prime \prime }$ is the average heat flux and *l* is the length of the interface curve or the droplet baseline.

**Figure 12:**
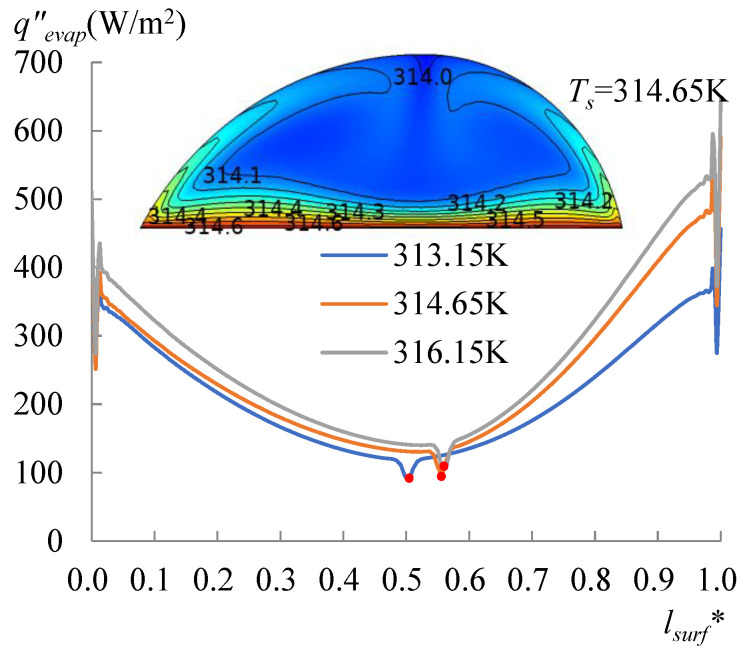
The heat flux due to the evaporation occurred at the LA interface for *T_s_* = 313.15 to 316.15 K. The lowest point moved rightwards with the increasing heating temperature.

**Figure 13:**
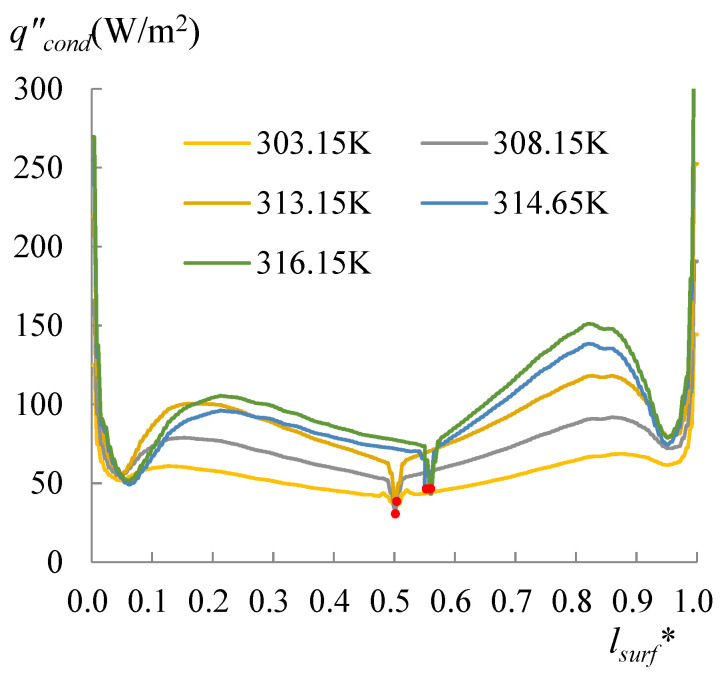
The heat flux conducted from droplet surface to the air for *T_s_* = 303.15 to 316.15 K. As well as the movement of the position of the lower heat flux, it also shows that the heat flux was higher on the right side than on the left side.

**Figure 14:**
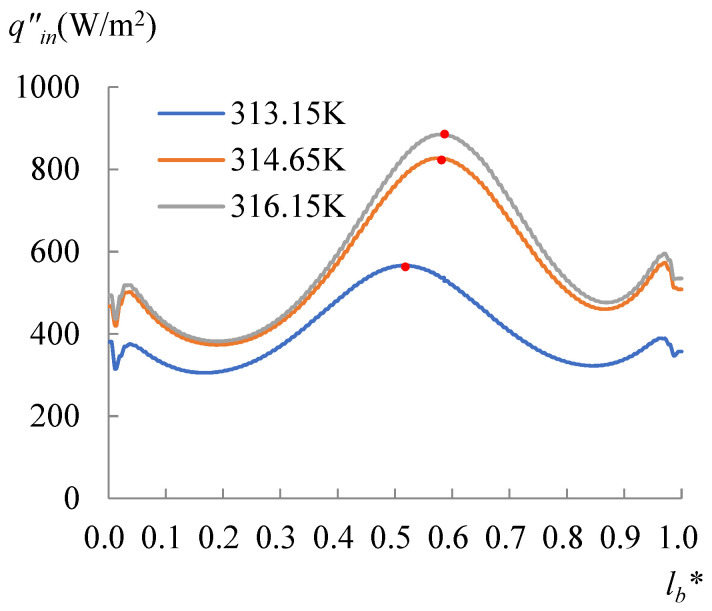
The heat flux conducted from the substrate for *T_s_* = 313.15 to 316.15 K. The higher heat flux (dividing points) moved rightward with the increasing heating temperature.

To specify the effect of the heating temperature on the movement of the dividing point or the formation of the single vortex, evaporation with *T_s_* between 313.65 and 318.15 K was particularly simulated as well. Curves of the heat flow lost by the evaporation for *T_s_* of 314.65 and 316.15 K are drawn in Figure 12. The corresponding heat flux conducted from the droplet to the air is illustrated in Figure 13, and the heat flux conducted from the substrate is drawn in Figure 14. As shown in these figures, compared with the curves of *T_s_* = 313.15 K, the dividing points for *T_s_* of 314.65 and 316.15 K moved rightward. The data with the *T_s_* ranging from 303.15 to 316.15 K were obtained for either side, separately. This was carried out by calculating Equation (21) using the heat flux shown in Figure 10, Figure 11, Figure 12, Figure 13 and Figure 14. Subsequently, substituting *q_evap_* and *q_cond_* into Equation (20), *q_out_* was also obtained, as shown in Figure 14. A useful parameter of Δ*q* can be found by:

(22) $$\Delta q=\left(q_{in}-q_{out}\right)|{}_{60{}^{\circ}}-\left(q_{in}-q_{out}\right)|{}_{78{}^{\circ}}$$

**Figure 15:**
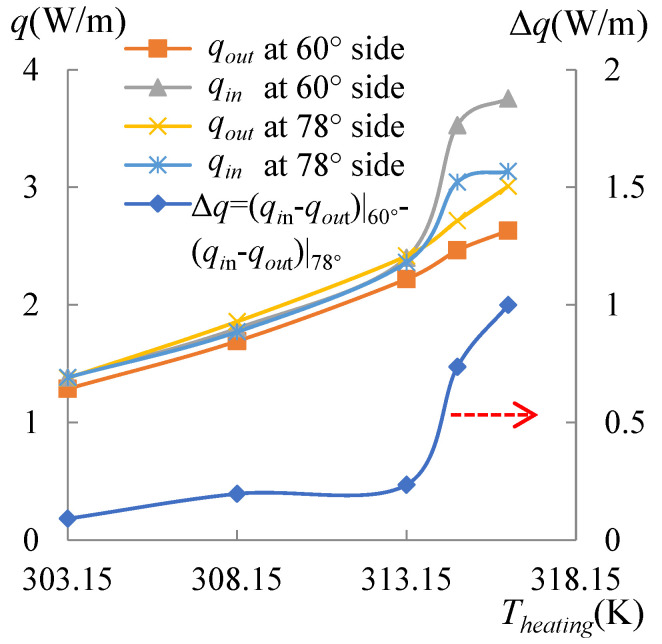
The input, output, and differential heat flow for *T_s_* = 303.15 to 316.15 K. There was a great jump in the input powers for both sides when *T_s_* = 313.15K. Furthermore, the differential heat flow indicating the power differential between both sides had a great jump as well.

Results calculated by Equation (22) are shown in Figure 15. It can be seen from Figure 15 that Δ*q* was increasing from 0.09 to 1.00 W/m with the heating temperature, indicating that the power left in the droplet at the side with CA of 60° was higher than the side with a CA of 78°, and the discrepancy was enhanced by the heating temperature. When the heating temperature rose over 313.15 K, the dividing point was driven toward the side with a CA of 78°, until the heating temperature reached 318.15 K, at which time the power at the side with smaller CA defeated the other side and a single vortex emerged. The *q_in_* and *q_out_* curves showed that when *T_s_* ≤ 313.15 K, the input power was similar for both sides and close to the output power; however, when *T_s_* > 313.15 K, the input power from the substrate dramatically increased, especially on the side with a CA of 60°.

### 3.2. Comparison with Spherical Droplets of 60° and 78°

**Figure 16:**
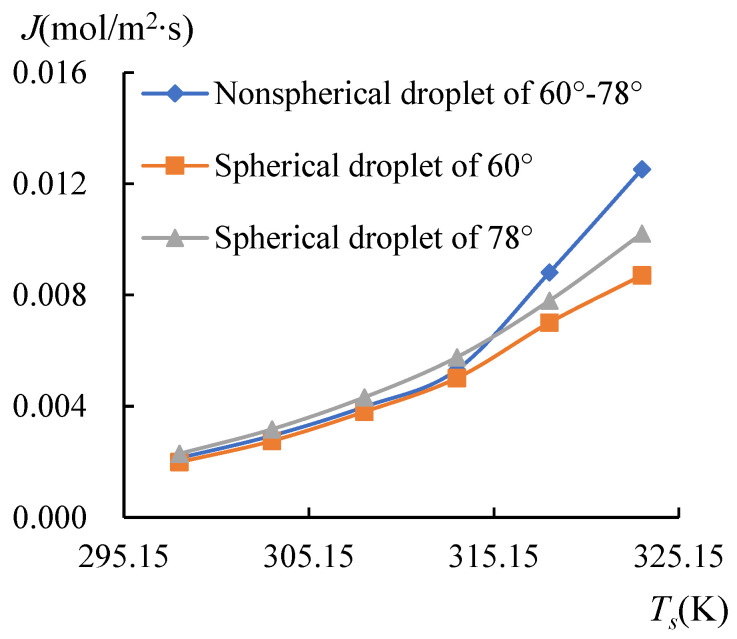
The evaporation flux of spherical and non-spherical droplets. For *T_s_* < 318.15 K, the evaporation flux of the non-spherical droplet (60°–78°) lies between the ones of spherical droplets (60° and 78°), however when *T_s_* ≥ 318.15 K, the non-spherical droplet surpasses spherical droplets.

Since the cross-section of the non-spherical droplets (60°–78°) is composed of parts from the spherical droplets (60° and 78°), a comparison was performed in this section to reveal the difference in the evaporation flux between these spherical droplets and the non-spherical droplet. The spherical droplets of 60° and 78° were simulated as evaporated at the heating temperature from 298.15 to 323.15 K. The curves of the average evaporation flux, *J,* from the LA interface of the droplets are drawn in Figure 16.

The evaporation flux of a spherical sessile droplet is proportional to its curvature, which can be deduced from Fick’s first law. The evaporative flux can be expressed as:

(23) $$J=-D\frac{dc}{dr}$$

where *dc*/*dr* is the vapor concentration gradient. The mass evaporated from the droplet could be written as:

(24) $$\frac{dm}{dt}=JA=DA\frac{dc}{dr}$$

where *A* is the surface area of a sessile spherical droplet, which is calculated using:

(25) $$A=2\pi r^{2}(1-\cos\theta )$$

By substituting Equation (23) into Equation (24), Equation (26) can be obtained as:

(26) $$\frac{dm}{dt}=-2\pi D(1-\cos\theta )r^{2}\frac{dc}{dr}=-2\pi rD(1-\cos\theta )\left(c_{sat}-c_{\infty }\right)$$

Then evaporation flux, *J,* can be calculated by:

(27) $$J=\frac{dm}{Adt}=-\frac{2\pi rD(1-\cos\theta )\left(c_{sat}-c_{\infty }\right)}{A}=-D\frac{c_{sat}-c_{\infty }}{r}$$

From Equation (27), the evaporation flux is proportional to the curvature expressed as 1/*r*; therefore, it can be found that the evaporation flux of the spherical droplet of 60° was smaller than the one of 78°. The non-spherical droplet is composed of the parts from the spherical droplets, so its curve rests between the two spherical droplets for *T_s_* ≤ 313.15 K, as shown in Figure 16. However, when the substrate was heated over 313.15 K, the temperature at the side with a CA of 60° was dramatically restored from the heated substrate, which was experimentally established. The evaporation flux was thus greatly enhanced on this side, thereby disintegrating the spherical droplets. The indication here is that the non-spherical droplet can surpass the spherical droplets in the evaporation flux when heated at a high temperature.

### 3.3. Contact Angle Discrepancy (CAD) Effect

**Figure 17:**
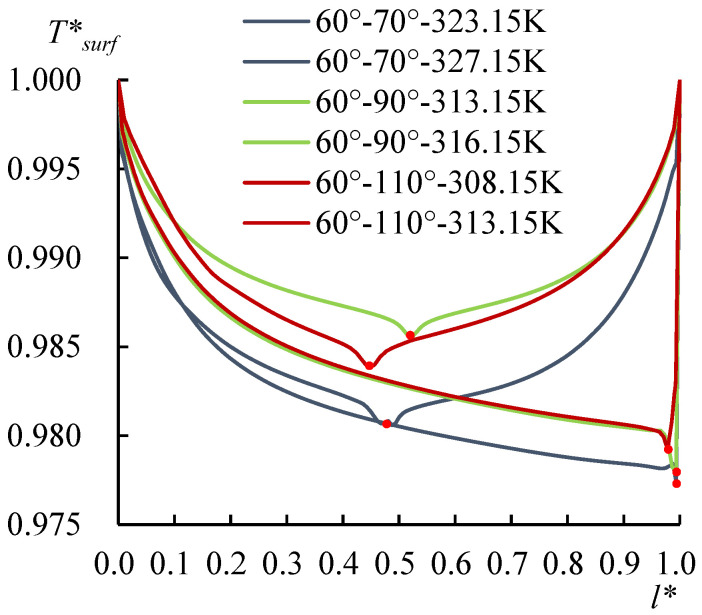
The temperature distribution along the LA interface of non-spherical droplets (60°–70°, 60°–90°, and 60°–110°). With the increase of the CA on the right side, the critical temperature for the formation of the single-vortex flow was reduced.

**Figure 18:**
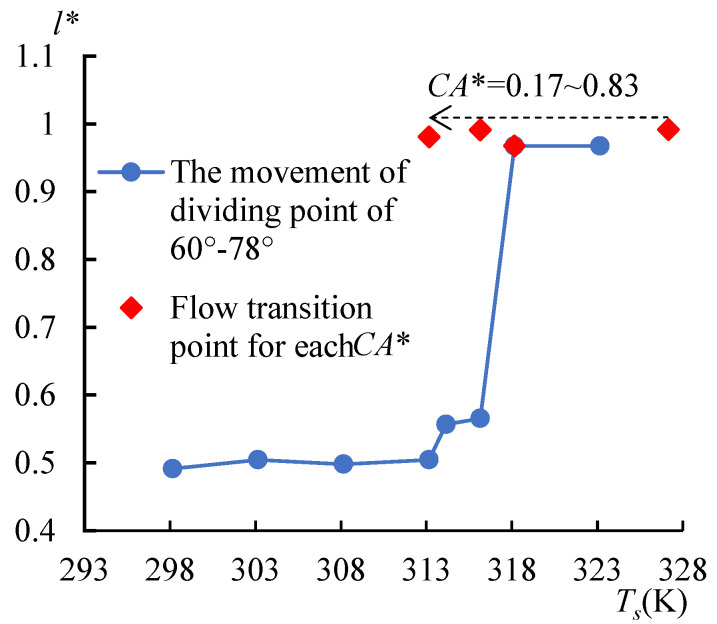
The position of dividing points vs. heating temperature. The diving points moved rightwards with the increasing *T_s_*. For the increased CA*, the single-vortex flow (transition point) formed at a lower heating temperature.

To investigate the CAD effect on the non-spherical droplet evaporation, three other non-spherical droplets whose contact angles on either side were 60°–70°, 60°–90°, and 60°–110° were simulated as well. The T*_surf_ curves in Figure 17 show the single-vortex flow for these droplets formed at 327.15, 317.15, and 313.15 K, respectively, indicated by the lowest surface temperatures reaching the right side of the droplet. As shown in Figure 18, the dividing points of the droplet at 60°–78° were pushed rightward (from l* = 0.50) when *T_s_* > 313.15 K, and the single-vortex flow formed at the temperature threshold of 318.15 K (l* = 0.97); however, for the non-spherical droplets with the increased CA* from 0.17 to 0.83 (the corresponding CA on the right side of the droplet was 70°, 78°, 90°, and 110°, respectively), which was calculated with the temperature threshold for the flow transition, was decreased. The reason could be analyzed as the direct relationship between the contact angle and the curvature of a sessile droplet. It is of difficulty for the droplet of higher curvature to recover its surface temperature, as concluded in Section 3.2. A higher CA* will enlarge the curvature difference, and the temperature recovery ability on both sides can be distinguished; thus, the dividing point is easier to be pushed toward the side with a larger CA and the temperature threshold for the flow transition can be reduced.

(28) $$CA*=\frac{\Delta CA}{CA}=\frac{CA_{right}-60{}^{\circ}}{60{}^{\circ}}$$

## 4. Conclusions

A 2D numerical model was presented to investigate the cross-section of non-spherical droplets, as well as the evolution of relevant parameters of surface temperature, velocity, and evaporation flux as they relate to the heating temperature. Compared to spherical droplets, non-spherical droplets showed some distinctive characteristics during heated evaporation:

(a) Instead of stabilizing on the apex of the droplet, the lowest surface temperature originally stayed near the middle of the liquid–air interface length and could be moved toward the higher curvature side via substrate heating.

(b) The lower curvature side was more sensitive to the substrate heating, resulting in the evaporation flux at this side showing improvement over the higher curvature side, so that non-spherical droplets could release more evaporation flux than spherical droplets.

(c) Single-vortex flow formed when the heating temperature was high enough, and it could be encouraged by the contact angle discrepancy.

## Data Availability

Not applicable.

## Nomenclature

*c*	molar concentration, mol/m^3^
*CA*	contact angle
*C_p_*	specific heat at constant pressure, J/(kg·K)
*D*	diffusion coefficient, m^2^/s
*f_st_*	force per unit area due to surface tension, N/m^2^
*h*	height of a droplet, m
*h_lv_ *	latent heat of evaporation, J/kg
*J*	evaporation flux, mol/(m^2^·s)
*k*	thermal conductivity, W/(m·K)
*l*	length of liquid–air interface
*m*	local evaporation flux, kg/(m^2^·s)
***n***	normal vector
*M_r_*	molar mass, kg/mol
*p*	pressure, Pa
*q*	heat flow, W/m
*q* *″*	heat flux, W/m^2^
*r*	radius, m
*R*	universal gas constant, J/(mol·K)
***t***	tangential vector
*u*	velocity in *x* direction, m/s
*v*	velocity in *y* direction, m/s

## Greek Symbols

*μ*	dynamic viscosity, Pa·s
*θ*	contact angle, °
*ρ*	density, kg/m^3^
*σ*	surface tension, N/m
*τ*	stress tensor, N/m^2^

## Subscripts

*av*	average
*b*	base
*c*	curvature
*cond*	conduction
*evap*	evaporation
*l*	liquid
*s*	substrate
*sat*	saturated condition
*surf*	surface of droplet
*v*	vapor
∞	environmental condition
